# Platform switching on wide-diameter external hex implants: a finite element analysis

**DOI:** 10.4317/jced.50991

**Published:** 2013-04-01

**Authors:** Stefano Sivolella, Riccardo Guazzo, Eriberto Bressan, Mario Berengo, Edoardo Stellini

**Affiliations:** 1 DDS, Clinical Assistant, Department of Oral Surgery, Padova University, Institute of Clinical Dentistry, Padova, Italy; 2 DDS, Resident, Department of Oral Surgery, Padova University, Institute of Clinical Dentistry, Padova, Italy; 3 DDS, Associate Professor, Department of Periodontology, Padova University, Institute of Clinical Dentistry, Padova, Italy; 4 MD, DDS, Professor and Chairman, Department of Oral Surgery, Padova University, Institute of Clinical Dentistry, Padova, Italy

## Abstract

Objectives: The objective of this work was to use finite element analysis to compare the effect of forces coming to bear on abutments 4.1 or 5.0 mm in diameter connected to a 5.0 mm implant (i.e. with or without platform switching). Study design: A 3D CAD model of a 5 x 11.5 mm external hex implant was developed, complete with a connection screw and either of two abutments, one 4.1 and the other 5 mm in diameter, to assess the influence of two loading conditions, i.e. 200 N loaded either axially or off center on the top of the abutment. Results and conclusions: In the symmetrically loaded models, greater stresses were transmitted to the bone in the area below the neck of the implant in the case of the wider-diameter abutment. When the narrower abutment was considered, the stress lines remained confined to the metal and were transferred to the bone in a more distal position. When the stresses in the bone where compared under non-symmetrical loading of the larger- and smaller-diameter abutments, the stresses reached lower values in the latter case. These findings indicate that platform switching (i.e. coupling a 4.1 mm abutment with a 5 mm implant) achieves a better, more even distribution of the peri-implant stresses deriving from simulated occlusal loads on the bone margins.

** Key words:**Platform switching, finite element analysis, implant.

## Introduction 

The term ‘platform switching’ refers to the use of an abutment narrower than the corresponding implant’s platform. Radiographic studies in 5.0 and 6.0 mm implants combined with 4.1 mm abutments in diameter have expectedly demonstrated smaller changes in terms of vertical marginal bone resorption than those occurring around implants with abutments of the same diameter (1). Numerous factors have been assessed to justify this phenomenon. From the biomechanical standpoint, platform switching seems to create more favorable conditions for the distribution of the load (2,3). It has been suggested that the biological processes taking place around the implant after the second surgical step (i.e. the insertion of the healing screw and the prosthetic abutment) differed when the external angle of the implant-abutment interface shifted inwards, further away from the external angle of the implant platform (1). The role of the microgap at the implant-abutment interface in causing bone resorption has also been considered, based on the assumption that this microgap contains fluids, molecules (disaccharides and small peptides), bacteria and inflammatory cells associated with the osteoclast activation that leads to peri-implant bone tissue resorption (2,4). Another factor linked to the effectiveness of platform switching in reducing marginal bone resorption concerns the establishment of the necessary biological width (the required dimension of the barrier of soft tissue consisting of junctional epithelium with an area of connective tissue). The biological width is determined physiologically and dimensionally stable for natural teeth and, likewise, for implants. Without enough of this peri-implant soft tissue to assure the biological width, it has been demonstrated that bone resorption will occur so that an adequate coupling and biological width can be restored (5,6). The three-dimensional morphology of the “cuff” of soft tissue around an implant depends on the diameter of the implant and on the design of the platform (7).

The aim of the present study was to use finite element analysis (FEA) to compare the effect of forces coming to bear on abutments of different diameters (4.1 mm and 5.0 mm) attached to a 5.0 mm implant in diameter inserted in a bone matrix.

## Material and Methods

The implant system studied comprised a 5 x 11.5 mm implant of the Osseotite® Biomet 3i type (Biomet 3i, Palm Beach, FL, USA), a Gold-Tite Hexed UniScrew connection screw (Biomet 3i), and two GingiHue Post abutments (Biomet 3i), one 4.1 and the other 5 mm in diameter.

First the real dimensions of the components were recorded using a gauge and an optical microscope. Then the 3D CAD model was prepared with the Rhino 3.0 solid modeling tool (Robert McNeel & Associates, Seattle, USA).

The complexity of the shapes involved and the calculation demands prompted us to adopt a few reasonable simplifications as follows: the thread on the connection screw was disregarded, while the thread on the implant was modeled, although it was abruptly interrupted; the bone was modeled using a simplified shape, i.e. a homogeneous and isotropic cylinder in which the implant was embedded up to the neck; and the implant was assumed to be perfectly osteointegrated.

After designing the shape of the two models (with and without platform shifting, PS), the finite element mesh was developed and applied, using a tetrahedron with 10 nodes of variable size, i.e. smaller in the areas where the greatest stresses were presumably concentrated.

All the numerical simulations were completed using ABAQUS/Standard FEA software (ABAQUS Inc., Paw-tucket, RI, USA). The mechanical properties of the bone and implant components studied were drawn from the literature (8). Two loading conditions were considered: (i) an axial load of 200 N coming to bear on the top of the abutment; and (ii) an off-centered load of 200 N, parallel to the axis of the implant and concentrated on a node beyond the top of the abutment.

Three different values were considered for the bone’s modulus of elasticity (8, 17, 34 GPa).

## Results 

- Symmetrical loading models

The model reached convergence and solved the problem in all the cases analyzed.

The chromatic maps used to compare the results represent the values of the Von Mises equivalent stresses in the various components of the system.

**Figure 1:**
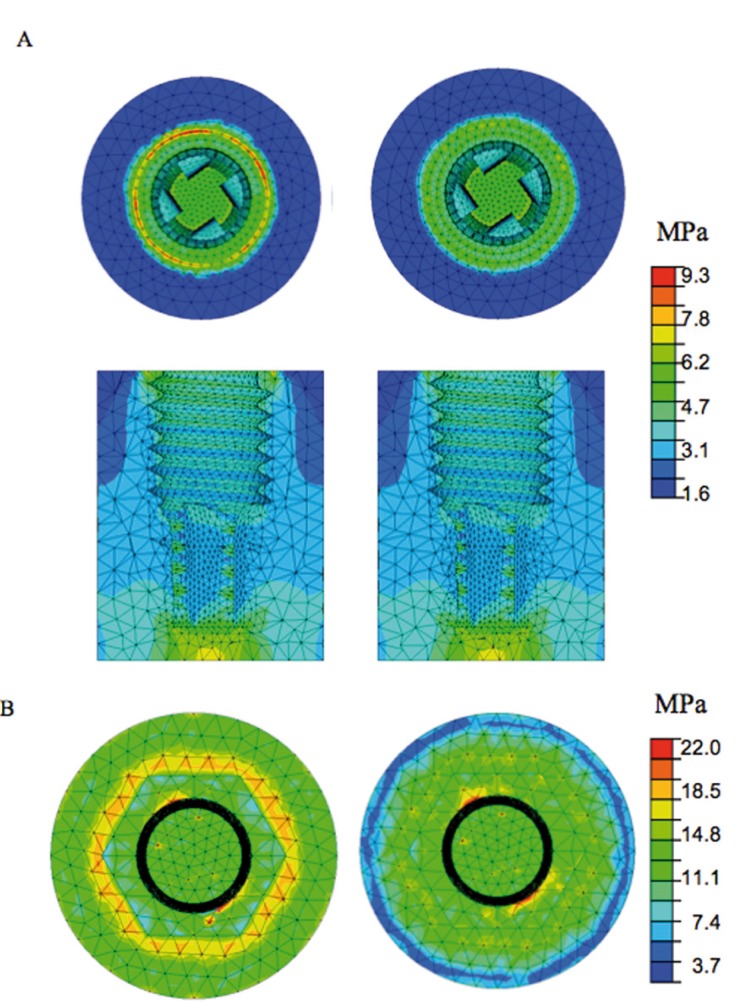
A. Distribution of Von Mises stresses on bone in the case of a large (left) and small (right) abutment. The images show a longitudinal section of the bone under symmetrical loading; B. Distribution of Von Mises stresses on the upper portion of an implant in direct contact with a symmetrically loaded large (left) and small (right) abutment.

In the bone, the distribution of the stresses was very similar in the two cases considered (Fig. 1). In particular, the greatest stresses occurred in line with the area of bone under the neck of the implant and at its base. It was clear, however, that the highest stresses were transmitted to the bone under the neck of the implant in the case of the larger-diameter abutment, whereas in the situation with a narrower abutment, the stresses in the implant were highest in line with the first turns of the thread (Fig. 1).

The stress lines followed a different trend in the two cases. With the wider abutment, the stresses were distributed over a broader surface area of the neck and consequently came to bear most on the underlying bone. With the narrower abutment, the stress lines remained confined within the metal and were transferred to the bone in a more distal position.

**Figure 2:**
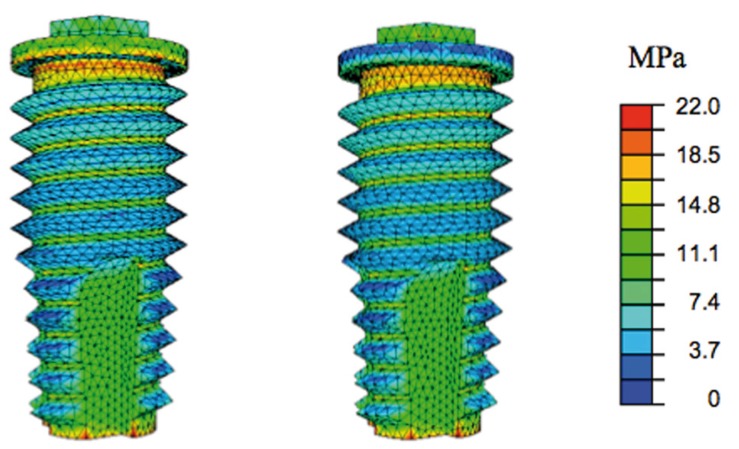
Distribution of Von Mises stresses on the implant under symmetrical loading with large (left) and small (right) abutment.

The distribution of the stresses and the values reached in the remainder of the implant were practically the same (Fig. 2).

As for the abutment, the stresses on the top were comparable in the two cases (wider and narrower abutments), but the stresses increased in the narrower abutment where the load-supporting cross-section (the area in which the shape of the abutment actually changed) was smaller.

The connection screw proved to be of no particular interest. At the top, where it was coupled with the abutment with some degree of slack, there were no stresses coming to bear because the screw was completely unloaded at the time of the structure’s compression (apart from a minimal component due to friction between the two parts).

- Non-symmetrical loading models 

**Figure 3:**
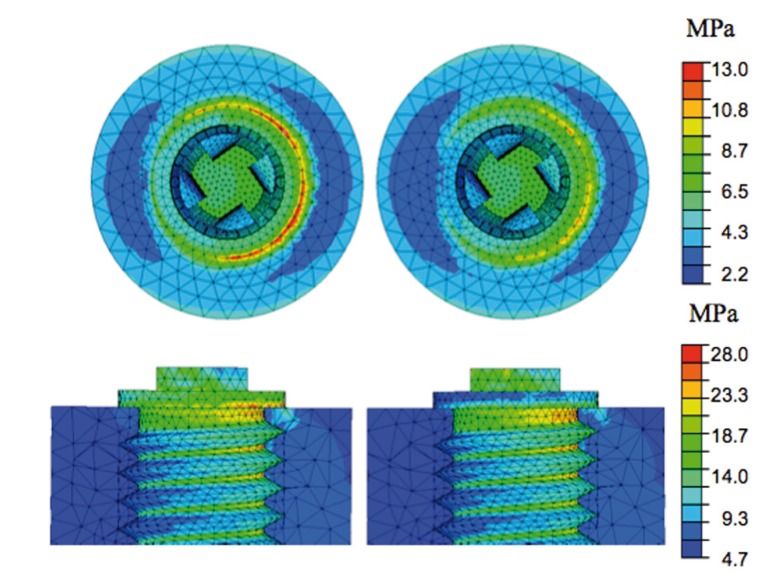
Von Mises equivalent stresses on the bone and the implant wall in the case of a large (left) and small (right) abutment under non-symmetrical loading conditions.

These simulations were used to study the effect of varying the load on the bone with the two different sizes of the abutment, while the mechanical characteristics of the material remained the same as in the previously analyzed case. In particular, a 200 N load was applied off center vis-à-vis the abutment. The forces inside the bone as a result of shifting the load coming to bear on the end of the abutment were essentially a bending moment and an axial compressive action. Being equivalent stresses, the Von Mises stresses can be used in this case to give a general indication of the stresses coming to bear on the bone for the purposes of a comparison between the two different abutments (Fig. 3). The stresses were not evenly distributed over the cross-section of the bone because of the non-symmetrical loading condition. The stress values reached were higher than in the case of a concentric load. From the comparison between the stresses inside the bone when a wider or narrower abutment was used, it emerged clearly that the stresses reached lower values in the latter case. The greatest difference between the maximum stresses reached in the two cases coincided with the neck of the implant. This is due to the different shaping of the abutments, which transmitted the loads in a different way.

- Model with different bone properties

Two further analyses were conducted on the implant modeled with the wider abutment to shed more light on how the bone’s modulus of elasticity could influence its behavior.

While the previously-described simulations were all performed assuming a modulus of elasticity of 17 GPa for the bone, these additional analyses were conducted considering a lower modulus of elasticity for the bone (E = 8 GPa), and a higher one (E = 34 GPa). The characteristics of the other materials involved in the numerical model remained the same.

It was clear from the comparison between the three models that the Von Mises stresses coming to bear on the proximal portion, underneath the neck, were greater in the case of a higher modulus of elasticity (i.e. a more brittle bone). In the distal portion (near the connection at the base), the stresses followed the opposite trend in the three cases considered, because the stresses are transferred entirely to the bone when the implant no longer sustains them.

## Discussion

Our finite element analyses (FEA) demonstrated that platform switching coincides with a distribution of the stress further away from the bone-implant interface at cervical level, while the stress is more concentrated on a level with the crown, abutment, connection screw and implant-abutment connection (3,9,10).

Some authors (8,11) came to the conclusion, consistently with our findings, that platform switching reduced the main Von Mises compressive and tensile stresses in compact bone by comparison with the conventional solution, while these stresses increased in the spongy bone. In other words, platform switching shifts the stresses from the area of compact bone to the area of spongy bone under oblique loading conditions (8). Canullo et al. (2) also found that platform switching enabled a more homogeneous distribution of the stresses along the length of the implant.

Maeda et al. (10) conducted a study on a 4 x 15 mm implant modeled with no thread, embedded in a bone matrix with a Young’s modulus of 15 GPa for the cortical bone and 1.5 GPa for the medullary bone, connected to abutments 4 and 3.25 mm in diameter, with a non-symmetrical 10N load brought to bear on them. The authors demonstrated that displacing the concentration of the stresses further away from the bone-implant interface is one of the advantages of PS.

Pessoa et al. (12), and Calvo-Guirado et al. (13) found no significant improvement in the mechanical behavior of implants associated with narrower abutments. According to these authors, accurate control over functional loading and an adequate intra-osseous stability are more important, from a biomechanical standpoint, to the long-term survival of the implant.

Other factors have been analyzed with a view to justifying the efficacy of PS, including: the shifting further away of the microgap between the implant and the abutment; the implant’s diameter; the shape of the coronal portion of the implant (with or without threading); the type of surface finish; the type of implant-abutment connection; and, from the clinical standpoint, the gingival biotype and any presence of adjacent teeth (11,12,14).

Vigolo and Givani (15) reported on their clinical application of PS in much the same conditions as those as-sumed for this FEA. A year after making the implant-abutment connection, they found significant differences in terms of the loss of marginal bone with a better result in the PS group than in the group with abutments of the same diameter as the implant platform. In subsequent years the differences were no longer significant, however.

Peri-implant vertical bone loss was also seen in cases in which PS had been applied, but it was minimal in the majority of cases (16,17), and always more limited than in cases in which an abutment of the same diameter as the implant had been used (18); in the former, this enabled the peri-implant bone level to be preserved (17) and improved the long-term predictability of the implant treatment (19).

Wagenberg and Froum (20) assessed 94 implants completed with PS after a follow-up of at least 11 years: 75.5% of the implants showed no signs of medial bone loss, and 71.3% showed no distal bone loss. The amount of bone loss was less than 0.8 mm at more than 84% of the medial surfaces and 88% of the distal surfaces. The authors consequently emphasized that PS helps to prevent crestal bone loss and obtain satisfactory cosmetic results (21).

Atieh et al. (22) reported that the survival rate of implant involving PS was comparable with that of non-PS implants. There was a significantly smaller loss of marginal bone around PS implants, and a difference between the implant and abutment diameters of at least 0.4 mm was associated with a better bone response. The quantity of marginal bone resorption was inversely proportional to the difference between the diameters of the implant and abutment (22-24). In addition to a better distribution of the stresses at the bone-implant interface, this may be due to the fact that the PS technique increases the distance between the inflammatory cell infiltrate associated with the abutment and the marginal bone, consequently limiting its contribution to causing bone resorption. There is also a reduction in the amount of bone that must necessarily be reabsorbed to expose the minimum quantity of implant surface area to allow for soft tissue integration.

Canullo et al. (25) assessed the peri-implant microbial flora in cases of traditional implants and PS implants, finding no differences between the two conditions and ruling out any influence of this factor on the different amounts of bone resorption. Even histology identified no differences in the composition of the peri-implant soft tissues around implants performed with or without PS, despite X-ray evidence of different levels of marginal bone resorption (26).

On the other hand, some clinical studies published in the literature found no differences in the loss of vertical bone height around implants performed with or without PS (27,28).

Finally, the use of FEA in implantology demands the use of certain assumptions (simplifications) that have to be taken into account when it comes to interpreting the results (29). These simplifications concern the shape of the model, the properties of the materials, the boundary conditions, and the interface between bone and implant (8,29,30). In the light of all the considerations, the debate on how the use of PS affects the preservation of peri-implant tissues remains open.

## Conclusions

Despite the limits of this study, the results obtained indicate that using an abutment 4.1 mm in diameter coupled with a 5 mm implant leads to a better, more homogeneous distribution of the stresses deriving from simulated occlusal loads on the marginal bone around the implant. This is true for symmetrical, axial loads and also in the case of non-symmetrical, off-center loads, supports the use of the PS technique with a view to reducing peri-implant marginal bone resorption.

Our results are consistent with other reports in the literature, in which a majority of the authors who conducted experimental and clinical studies on this issue found significant advantages deriving from the application of the platform switching method.

Further studies will be needed to clarify the biological process correlating platform switching with the preservation of the hard and soft peri-implant tissues.
